# Correction: Ratka et al. The Effect of In Vitro Electrolytic Cleaning on Biofilm-Contaminated Implant Surfaces. *J. Clin. Med.* 2019, *8*, 1397

**DOI:** 10.3390/jcm11030882

**Published:** 2022-02-08

**Authors:** Christoph Ratka, Paul Weigl, Dirk Henrich, Felix Koch, Markus Schlee, Holger Zipprich

**Affiliations:** 1 Department of Prosthodontics, Goethe University, 60590 Frankfurt am Main, Germany; c.ratka@gmx.de (C.R.); weig@em.uni-frankfurt.de (P.W.); 2 Department of Trauma, Hand & Reconstructive Surgery, Goethe University, 60590 Frankfurt am Main, Germany; d.henrich@trauma.uni-frankfurt.de; 3 Private Practice, Department of Maxillofacial Surgery, Goethe University, 60590 Frankfurt am Main, Germany; felixpkoch@gmx.de (F.K.); markus.schlee@32schoenezaehne.de (M.S.)

There was an error in the original article [1]. **A brand name was inadvertently used for a general method. Instead of the correct acronym “PSS”, the acronym “AirFlow” was written.**

A correction has been made to **Section 2.13. Statistics, 2nd Paragraph, last sentence:**

**Please change the acronym “AirFlow” into “PSS”:**

**Comparisons between the surface and material groups for PSS were performed with the Kruskal–Wallis test.**

The authors apologize for any inconvenience caused and state that the scientific conclusions are unaffected. The original article has been updated.
